# CMAB009 plus irinotecan versus irinotecan-only as second-line treatment after fluoropyrimidine and oxaliplatin failure in *KRAS* wild-type metastatic colorectal cancer patients: promising findings from a prospective, open-label, randomized, phase III trial

**DOI:** 10.1186/s40880-019-0374-8

**Published:** 2019-05-24

**Authors:** Yuankai Shi, Jin Li, Jianming Xu, Yan Sun, Liwei Wang, Ying Cheng, Wei Liu, Guoping Sun, Yigui Chen, Li Bai, Yiping Zhang, Xiaohui He, Yi Luo, Zhehai Wang, Yunpeng Liu, Qiang Yao, Yuhong Li, Shukui Qin, Xiaohua Hu, Feng Bi, Rongsheng Zheng, Xuenong Ouyang

**Affiliations:** 1 Department of Medical Oncology, Beijing Key Laboratory of Clinical Study On Anticancer Molecular Targeted Drugs, National Cancer Center/National Clinical Research Center for Cancer/Cancer Hospital, Chinese Academy of Medical Sciences and Peking Union Medical College, Beijing, 100021 P. R. China; 2 Fudan University Shanghai Cancer Center, Shanghai, 200032 P. R. China; 3 The Affiliated Hospital of Military Medical Sciences, Beijing, 100071 P. R. China; 4 Shanghai General Hospital, Shanghai, 200080 P. R. China; 5 Jilin Cancer Hospital, Changchun, 130012 Jilin P. R. China; 6 Tumor Hospital of Hebei Province, Shijiazhuang, 050011 Hebei P. R. China; 7 The First Affiliated Hospital of Anhui Medical University, Hefei, 230022 Anhui P. R. China; 8 Fujian Provincial Cancer Hospital, Fuzhou, 350014 Fujian P. R. China; 9 Chinese People’s Liberation Army General Hospital, Beijing, 100853 P. R. China; 10 Zhejiang Cancer Hospital, Hangzhou, 310022 Zhejiang P. R. China; 11 Hunan Cancer Hospital, Changsha, 410013 Hunan P. R. China; 12 Shandong Cancer Hospital, Jinan, 250117 Shandong P. R. China; 13 The First Hospital of China Medical University, Shenyang, 110001 Liaoning P. R. China; 14 Tianjin People’s Hospital, Tianjin, 300121 P. R. China; 15 Sun Yat-sen University Cancer Center, State Key Laboratory of Oncology in South China, Collaborative Innovation Center of Cancer Medicine, Guangzhou, 510060 Guangdong P. R. China; 16 Chinese People’s Liberation Army Bayi Hospital, Nanjing, 210002 Jiangsu P. R. China; 17 The Guangxi Zhuang Autonomous Region Tumor Hospital, Nanning, 530021 Guangxi P. R. China; 18 West China Hospital, Chengdu, 610041 Sichuan P. R. China; 19 First Affiliated Hospital of Bengbu Medical College, Bengbu, 233004 Anhui P. R. China; 20 Fuzhou People’s Liberation Army General Hospital, Fuzhou, 350025 Fujian P. R. China

**Keywords:** CMAB009, Cetuximab, Irinotecan, Second-line, mCRC, EGFR, *KRAS*, Immunogenicity, Fluoropyrimidine, Oxaliplatin failure

## Abstract

**Background:**

The 5**-**fluorouracil/leucovorin plus oxaliplatin (FOLFOX) regimen is the standard first-line treatment for metastatic colorectal cancer (mCRC), however, the optimal second-line regimen for *KRAS* wild-type mCRC patients is still investigational. In this study, we aimed to determine the clinical efficacy and safety of CMAB009 plus irinotecan compared to irinotecan-only as a second-line regimen for treating *KRAS* wild-type mCRC patients.

**Methods:**

Patients with *KRAS* wild-type mCRC who had previously failed to respond to FOLFOX treatment were randomly assigned in a 2:1 ratio, to receive CMAB009 plus irinotecan or irinotecan-only. Patients receiving irinotecan-only were permitted to switch to CMAB009 therapy on disease progression and were grouped as the sequential-CMAB009 arm. The primary endpoints were overall response rate (ORR) and median progression-free survival (PFS). The secondary endpoints were median overall survival (OS), disease control rate (DCR), clinical benefit rate (CBR), and duration of response (DOR).

**Results:**

The CMAB009 plus irinotecan arm demonstrated significantly improved ORR (33.2% vs. 12.8%; *P* < 0.001) and longer median PFS (169 days vs. 95 days; *P* < 0.001) as compared to the irinotecan-only arm. Patients receiving CMAB009 plus irinotecan also demonstrated improved DCR (80.1% vs. 65.2%, *P* < 0.001), CBR (30.0% vs. 14.6%, *P* < 0.001), and DOR (210 days vs. 109 days; *P *< 0.001) as compared to irinotecan-only. However, patients treated with CMAB009 had an increased risk of skin rash (66.9% vs. 5.5%, *P* < 0.001) and paronychia (9.8% vs. 0.0%, *P* < 0.001). Anti-drug antibodies (ADA) were detected in 3.6% of patients, and only 0.9% of patients who received CMAB009 experienced hypersensitivity reactions. In patients receiving sequential-CMAB009 therapy after failure with irinotecan, their median PFS was 84 days (95% CI 65 to 113 days). The median OS was 425 days for patients receiving CMAB009 plus irinotecan and 401 days for those with sequential-CMAB009 (*P *= 0.940).

**Conclusions:**

Treatment with CMAB009 plus irinotecan was found to be a superior second-line regimen in comparison to irinotecan-only in *KRAS* wild-type mCRC patients. Further, switching to CMAB009 can be considered as an efficient third-line of treatment after treatment failure with second-line irinotecan-only.

*Trial registration* ClinicalTrials.gov: NCT01550055, retrospectively registered on March 9, 2012.

## Background

Colorectal cancer (CRC) is one of the most common malignant tumors, with an estimated 1.4 million new cases and nearly 700 thousand cancer deaths reported worldwide in the year 2012 [1]. The incidence and mortality rates of colorectal cancer rank fifth in China [2], and the incidence is still rising [3]. The prognosis of metastatic CRC (mCRC) is poor with a 5-year overall survival (OS) rate < 15% [4, 5]. Patients with unresectable mCRC receiving supportive care alone have been shown to have a poor prognosis, with a median OS of 5 months [6]. By contrast, the 5-year OS rate of patients with unresectable mCRC who received first-line chemotherapy with 5-fluorouracil (5-FU)/leucovorin (LV) plus oxaliplatin (FOLFOX) was 10% [7].

The FOLFOX regimen has become the standard first-line therapy for the treatment of unresectable mCRC [8–11]. Other recommended first-line chemotherapy regimens include capecitabine plus oxaliplatin (CapeOX), FOLFOX plus bevacizumab, CapeOX plus bevacizumab, and FOLFOX plus cetuximab (*KRAS*/*NRAS* wild type only) [12, 13]. However, after the failure with first-line therapy, which combination chemotherapy regimen would be the optimal second or third-line treatment is yet to be confirmed, as such strategic trials investigating these are urgently needed.

CMAB009, a recombinant, human/mouse chimeric monoclonal antibody (mAb) specifically targeting the human epidermal growth factor receptor (EGFR), competitively inhibits ligand-binding and interrelated downstream signaling. It has the same amino acid sequence as ERBITUX^®^ (cetuximab), but slightly different abilities for glycosylation and other post-translational modifications (PTMs). CMAB009 is expressed by the Chinese hamster ovary (CHO) cells while cetuximab is expressed by the mouse cell line SP2/0 which also expresses the gene for α-1,3-galactosyltransferase [14]. In most patients who have developed a hypersensitivity reaction to cetuximab, IgE antibodies against the cetuximab were found to be already present in their serum prior to the start of the therapy [14]. These antibodies were found to be specific for galactose-α-1,3-galactose (Gal (α 1-3) Gal). Since CHO cells do not produce α-1,3-galactosyltransferase, they have a pattern of glycosylation that differs from that of SP2/0 [14–16], as such, CMAB009 expressed in CHO cells has a lower level of Gal (α 1-3) Gal-containing glycans [15]. This suggests that CMAB009 might have lower immunogenicity and reduced hypersensitivity reactions as compared to cetuximab.

In our previous retrospective study, we have shown that CMAB009 demonstrated good efficacy and acceptable tolerance in patients with chemotherapy-resistant advanced CRC [17]. In this study, we aimed to prospectively determine the clinical efficacy and safety of CMAB009 plus irinotecan as compared to that of irinotecan-only in *KRAS* wild-type mCRC patients who had treatment failure with first-line FOLFOX regimen.

## Patients and methods

### Patient selection

This prospective, open-label, randomized, phase III trial was conducted at 38 centers in China (Table 1). Patients were eligible if they had previous documented treatment failure (disease progression or discontinuation due to toxicity) with FOLFOX regimen for histologically confirmed mCRC and had wild-type *KRAS* mutation. Other inclusion criteria were: age between 18 and 70 years, an Eastern Cooperative Oncology Group (ECOG) performance status (PS) score of 0 or 1, a life expectancy of more than 3 months starting from the time of enrollment, no other malignant tumors, except for patients who had been cured for cervical carcinoma in situ, skin basal carcinoma, or squamous cell carcinomas. The exclusion criteria were: chemotherapy within 4 weeks prior to enrollment, abnormal serum hematologic function [hemoglobin (Hb) < 90 g/L; platelet count (PLT) < 100 × 10^9^/L; absolute neutrophil count (ANC) < 1.5 × 10^9^/L; or white blood cell count (WBC) < 4.0 × 10^9^/L), abnormal hepatorenal function (total bilirubin (TBIL), more than onefold higher than the upper limit of the normal range; blood urea estrogen (BUN) and creatinine (Cr), more than 1.5-fold higher than the upper limit of the normal range; or alanine aminotransferase (ALT) and aspartate aminotransferase (AST), more than fivefold higher than the upper limit of the normal range with hepatic metastases or more than 2.5-fold higher than the upper limit of the normal range without hepatic metastases], serious cardiac insufficiency, known history of brain metastases, and prior therapy with EGFR-targeting agents. Women who were pregnant or breastfeeding were also excluded.

**Table 1:** Eligible *KRAS* wild-type patients were identified at 38 hospitals in China

Participating institutions	Principle investigator in each institution	No. of patients
Cancer Hospital, Chinese Academy of Medical Sciences and Peking Union Medical College	Yuankai Shi	18
Fudan University Shanghai Cancer Center	Jin Li	42
The Affiliated Hospital of Military Medical Sciences	Jianming Xu	38
Shanghai General Hospital	Liwei Wang	30
Jilin Cancer Hospital	Ying Cheng	21
Tumor Hospital of Hebei Province	Wei Liu	22
The First Affiliated Hospital of Anhui Medical University	Guoping Sun	23
Fujian Provincial Cancer Hospital	Yigui Chen	24
Chinese PLA General Hospital	Li Bai	20
Zhejiang Cancer Hospital	Yiping Zhang	21
Hunan Cancer Hospital	Yi Luo	18
Shandong Cancer Hospital	Zhehai Wang	18
The First Hospital of China Medical University	Yunpeng Liu	18
Tianjin People’s Hospital	Qiang Yao	15
Sun Yat-sen University Cancer Center	Yuhong Li	14
Chinese PLA Bayi Hospital	Shukui Qin	12
The Guangxi Zhuang Autonomous Region Tumor Hospital	Xiaohua Hu	12
West China Hospital	Feng Bi	11
First Affiliated Hospital of Bengbu Medical College	Rongsheng Zheng	10
Fuzhou PLA General Hospital	Xuenong Ouyang	10
Peking Union Medical College Hospital	Chunmei Bai	10
Tianjin Medical University Cancer Institute & Hospital	Yi Ba	16
Jiangsu Cancer Hospital	Jifeng Feng	10
General Hospital of Jinan Military Region	Baocheng Wang	10
Chongqing General Hospital	Min Fu	9
The First Affiliated Hospital of The Third Military Medical University	Houjie Liang	7
Tongji Hospital of Tongji Medical College, Huazhong University of Science and Technology	Shiying Yu	7
Ruijin Hospital, Shanghai Jiaotong University School of Medicine	Jun Zhang	6
The Second Xiangya Hospital of Central South University	Chunhong Hu	6
No. 3 People Hospital Affiliated to Shanghai Jiaotong University School of Medicine	Bin Jiang	5
Chongqing Cancer Hospital	Ying Xiang	5
Nanfang Hospital	Rongwei Luo	5
The First Affiliated Hospital of Suzhou University	Min Tao	4
Affiliated Hospital of Nantong University	Guoxin Mao	4
Sichuan Provincial People’s Hospital	Honglin Hu	3
Gansu Provincial Cancer Hospital	Weihua Zhang	3
Xijing Hospital	Wenchao Liu	3
Kunming General Hospital of Chengdu Military Command	Hong Chen	2

The protocol of this study was approved by the ethics committee board at each center and all patients provided signed informed consent before participation.

### Study design

Patients were randomly assigned in a 2:1 ratio to receive either CMAB009 (Shanghai Zhangjiang Biotech Co., Shanghai, China) plus irinotecan (Qilu Pharma, Jinan, Shandong, China) or irinotecan-only, respectively. Patients receiving irinotecan-only could switch to CMAB009 therapy (labeled as the sequential-CMAB009 arm) upon diagnosis of disease progression.

The primary endpoints were overall response rate (ORR) and median progression-free survival (PFS). ORR was defined as the proportion of patients with a confirmed complete response (CR) or partial response (PR) according to response evaluation criteria in solid tumor (RECIST) version, 1.0. PFS was defined as the time from the date of entry into the trial to the date of first observed treatment failure (local and/or regional persistence/recurrence or distant metastasis) or death from any cause. The secondary endpoints were median OS (time from the date of entry into the trial to the date of death or the date the patient was last known to be alive), disease control rate [DCR, the duration of CR, PR, and stable disease (SD)], clinical benefit rate (CBR, defined as the sum of the number of patients who achieved CR, PR, and SD, and remained stable for a more than 24 weeks) and duration of response (DOR, time from the date of first evidence of CR or PR to the date of objective progression or the date of death due to any cause), and treatment safety.

### Treatment

Patients assigned to the CMAB009 plus irinotecan arm received an initial dose of CMAB009 at 400 mg/m^2^ intravenously over 2 h on day 1, and then 250 mg/m^2^ over 1 h weekly. Irinotecan, at a dosage of 180 mg/m^2^ intravenously, or 125–135 mg/m^2^ intravenously for those with prior pelvic/abdominal irradiation, was given over 90 min and was administered every 2 weeks in both treatment arms; starting more than 1 h after the CMAB009-infusion completion for patients in the CMAB009 plus irinotecan arm. Each treatment cycle lasted 2 weeks. The dosage of CMAB009 for patients in the sequential-CMAB009 arm was similar to that of the CMAB009 plus irinotecan arm. The treatments were continued until disease progression, unacceptable toxicity, or the patient withdrew consent.

The National Cancer Institute Common Terminology Criteria for Adverse Event Criteria (NCI CTCAE) version 3.0 was used to assess adverse events. The definition and grading of hypersensitivity reactions were based on documented symptoms list in the criteria, the characteristics of grade 1 reaction were transient flushing or rash; drug fever < 38 °C; those of a grade 2 reaction were rash; flushing; urticaria; dyspnea; drug fever ≥ 38 °C; and those of a grade 3 reaction were symptomatic bronchospasm, with or without urticaria; parenteral medication(s) indicated; allergy-related edema/angioedema; hypotension. Anaphylaxis and death were considered as a grade 4 and 5 reaction, respectively. CMAB009 was discontinued upon the occurrence of grade 3/4 hypersensitivity, after which the dose of irinotecan was to be reduced to 125–135 mg/m^2^ when grade 3/4 neutropenia, febrile neutropenia, thrombocytopenia, and leucopenia occurred. In the event of grade 4 nonhematologic toxicities (excluding diarrhea), both agents were discontinued.

### Assay to detect mutant *KRAS*

The tissue specimens (surgery or biopsy from the primary or metastatic tumor) of mCRC patients were evaluated at the central laboratory of the Chinese National Human Genome Center in Shanghai, and only patients with available KRAS mutational status at codon 12, 13 were included. Formalin-fixed, paraffin-embedded tumor sections were deparaffinized and air dried, and DNA was extracted using standard Proteinase K digestion and a DNeasy minispin column (Qiagen, Valencia, CA, USA). Mutant KRAS was detected using a validated DNA sequencing method that identifies seven somatic mutations located in codons 12 and 13 (Gly12Asp, Gly12Ala, Gly12Val, Gly12Ser, Gly12Arg, Gly12Cys, and Gly13Asp) using allele-specific real-time polymerase chain reaction at the central laboratory of The Chinese National Human Genome Center, Shanghai, China [18, 19].

### Response assessment

Measurable lesions were obtained at baseline (within the 4 weeks prior to the start of treatment) and evaluated every 6 weeks by computed tomography (CT) scans. Tumor response was assessed by local investigators based on the RECIST criteria version 1.0, until disease progression. After treatment completion, a follow-up assessment was conducted every 4 weeks, for up to 5 years after the last dose or until the patient succumbed or the last date of follow-up (July 23, 2015).

### Immunogenicity assessment

Blood samples were taken at week 0 (before CMAB009 infusion) and at 6, 12, 18, and 30 weeks after the first infusion, to determine the presence of ADA, which was analyzed using a competitive inhibition assay by the Surface Plasmon Resonance (SPR) (Shanghai Zhangjiang Biotechnology, Shanghai, P. R. China) while presence of neutralizing ADAs (NAb) were analyzed by competitive enzyme-linked immunosorbent assay (ELISA) (Shanghai Zhangjiang Biotechnology, Shanghai, P. R. China).

### Statistical analysis

At least 333 patients (CMAB009 plus irinotecan arm: 222 patients; irinotecan-only arm: 111 patients) were required to obtain a 90% power to detect an absolute difference in ORR. This in turn meant that after accounting for a typical study dropout rate of 10%, 495 patients (CMAB009 plus irinotecan: irinotecan-only, 330:165) were to be enrolled in the study to meet statistical requirements by satisfying the minimum number of patients outlined by the China Food and Drug Administration (CFDA, http://www.nmpa.gov.cn/WS04/CL2174/300629_9.html). An O’Brien and Fleming type α spending function was used to ensure an overall, two-sided, type I error rate of 5%. The ORR was compared between the treatment arms using a Cochran–Mantel–Haenszel test stratified by ECOG PS score (0 vs. 1). DCR, CBR, and DOR were assessed according to RECIST criteria, version 1.0. PFS and OS were analyzed by the Kaplan–Meier method. Primary comparisons between the treatment arms were made using a two-sided log-rank test stratified by ECOG PS. Hazard ratios with 95% confidence intervals (CI) were calculated from stratified Cox regression models with gender, age, and ECOG PS score.

## Results

### Patient characteristics

**Fig. 1:**
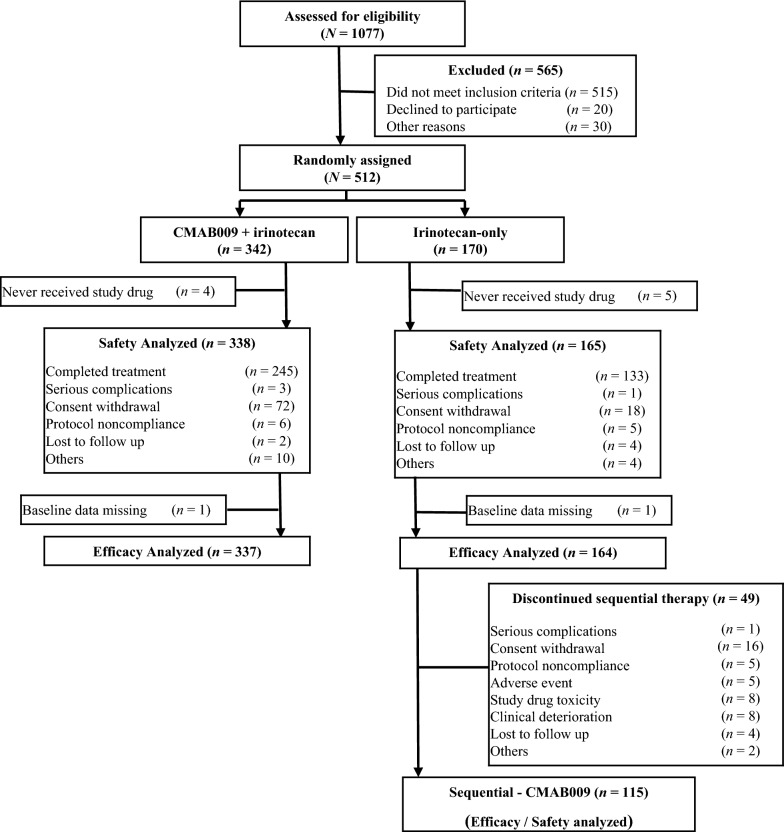
Flow chart illustrating the trial enrollment and patient outcomes

Between May 31, 2009, and September 23, 2011, a total of 1077 patients were assessed for eligibility. There were 35 patients with insufficient or poor-quality DNA samples. The observed *KRAS* mutation (codons 12 and 13) rate was 32.3% (337/1042). After exclusion of non-eligible patients, 512 *KRAS* wild-type patients, 342 in the CMAB0009 plus irinotecan arm and 170 in the irinotecan-only arm, were enrolled from 38 sites in China (Fig. 1, Table 1). The study arms were well balanced for clinical characteristics (Table 2).

**Table 2:** Baseline demographic and clinical characteristics of the 501 patients before the start of treatment

Characteristic^a^	CMAB009 plus irinotecan (*n *= 337)		Irinotecan-only (*n *= 164)		Entire study cohort (*n *= 501)		*P*
	No. of patients	%	No. of patients	%	No. of patients	%	
Age, years							0.652
Median	55.0		55.0		55.0		
Standard deviation	10.55		11.02		10.69		
< 65	287	85.2	141	86.0	428	85.4	
≥ 65	50	14.8	23	14.0	73	14.6	
Sex							0.246
Male	195	57.9	104	63.4	299	59.7	
Female	142	42.1	60	36.6	202	40.3	
Ethnic minority							0.121
Han	334	99.1	159	97.0	493	98.4	
Other	3	0.9	5	3.0	8	1.6	
ECOG performance status							0.120
0	136	40.4	56	34.1	192	38.3	
1	201	59.6	107	65.2	308	61.5	
2	0	0	1	0.6	1	0.2	
Previous therapy							
Chemotherapy	337	100	163	99.4	500	99.8	0.327
Radiation therapy	92	27.3	37	22.6	129	25.7	0.277
First-line therapy							0.667
Median duration, months	6		7		7		
Range, months	1–38		1–24		1–38		
Reason off therapy							0.552
Disease progression	301	66.8	144	63.7	445	65.8	
Adverse events	41	9.1	25	11.1	66	9.7	
Other	108	24.0	57	25.2	165	24.4	
Site of metastasis							0.873
Lung	142	42.1	73	44.5	215	42.9	
Peritoneum	25	7.4	17	10.4	42	8.4	
Liver	186	55.2	99	60.4	285	56.9	
Lymph node	106	31.5	60	36.6	166	33.1	
Other	105	31.2	50	30.5	155	30.9	
No. of disease sites							0.199
1	98	29.1	38	23.2	136	27.1	
≥ 2	239	70.9	126	76.8	365	72.9	

ECOG, Eastern Cooperative Oncology Group

^a^There was no significant difference in baseline patient characteristics between the 2 groups

### Treatment exposure

The median number of treatment cycles was 8 (1–80 cycles) for the CMAB009 plus irinotecan arm, 5 (1–27 cycles) for the irinotecan-only arm, and 4 (1–36 cycles) for the sequential-CMAB009 arm. The median irinotecan treatment duration was longer for the CMAB009 plus irinotecan arm (14.0 weeks; range 2.0 to 102.6 weeks) as compared to the irinotecan-only arm (10.0 weeks; range 2.0 to 53.2 weeks). In the CMAB009 plus irinotecan arm, the median CMAB009 treatment duration was 16.3 weeks (range 1.0 to 159.7 weeks). There were 115 patients who switched to CMAB009 treatment from the irinotecan-only therapy (sequential-CMAB009 arm) and the median treatment duration of CMAB009 was 7.4 weeks (range 1.0 to 72.0 weeks). The median dose intensity of irinotecan was higher in the irinotecan-only arm (97.6 mg/m^2^/week) than in the CMAB009 plus irinotecan arm (92.6 mg/m^2^/week). In the CMAB009 plus irinotecan arm, the median CMAB009 dose intensity was 263.3 mg/m^2^/week. For the sequential-CMAB009 arm, the median dose intensity was 286.4 mg/m^2^/week.

A dose modification of 35.0% (118/337) was recorded for irinotecan in the CMAB009 plus irinotecan arm and 20.1% (33/164) in the irinotecan-only arm. Dose modification for CMAB009 was 18.1% (61/337) in CMAB009 plus irinotecan arm and 12.2% (14/115) in the sequential-CMAB009 arm.

The safety analysis population consisted of patients who received at least one dose of the study drug and had at least one safety assessment after treatment administration (338 in the CMAB0009 plus irinotecan arm and 165 in the irinotecan-only arm). The efficacy analysis was performed in patients with at least one dose of the study drug and had complete baseline data (337 in the CMAB0009 plus irinotecan arm and 164 in the irinotecan-only arm) (Fig. 1).

### Treatment efficacy

#### Primary endpoints

The tumor response was evaluated in 501 investigated patients. The ORR was 33.2% (112/337) and 12.8% (21/164) in the CMAB009 plus irinotecan and irinotecan-only arms, respectively (*P* < 0.001, Table 3). For the sequential-CMAB009 arm, 13.9% (16/115) of the patients achieved PR and 49.6% (57/115) demonstrated SD.

**Table 3:** Therapeutic efficacies of CMAB009 plus irinotecan treatment versus irinotecan-only treatment

Treatment response	CMAB009 plus irinotecan (*n *= 337)		Irinotecan-only (*n *=164)		*P* ^*^
	No. of patients	%	No. of patients	%	
CR	4	1.2	1	0.6	
PR	108	32.0	20	12.2	
SD	158	46.9	86	52.4	
PD	47	13.9	44	26.8	
Not evaluable	20	5.9	13	7.9	
ORR^a^	112/337	33.2	21/164	12.8	< 0.001
95% CI of ORR	28.2–38.5		8.1–8.9		
DCR^b^	270/337	80.1	107/164	65.2	< 0.001
95% CI of DCR	75.5–84.2		57.4–72.5		
CBR^c^	101/337	30.0	24/164	14.6	< 0.001
95% CI of CBR	25.1–35.2		9.6–21.0		

CR, complete response; PR, partial response; SD, stable disease; PD, progressive disease; response classified by Response Evaluation Criteria in Solid Tumors (RECIST, version 1.0); ORR, overall response rate; DCR, disease control rate; CBR, clinical benefit rate

*Cochran–Mantel–Haenszel test stratified by Eastern Cooperative Oncology Group performance status (0 vs. 1) at random assignment

^a^Overall response either CR or PR

^b^Overall response CR PR or SD

^c^Overall response CR PR or SD, ≥ 24 weeks

**Fig. 2:**
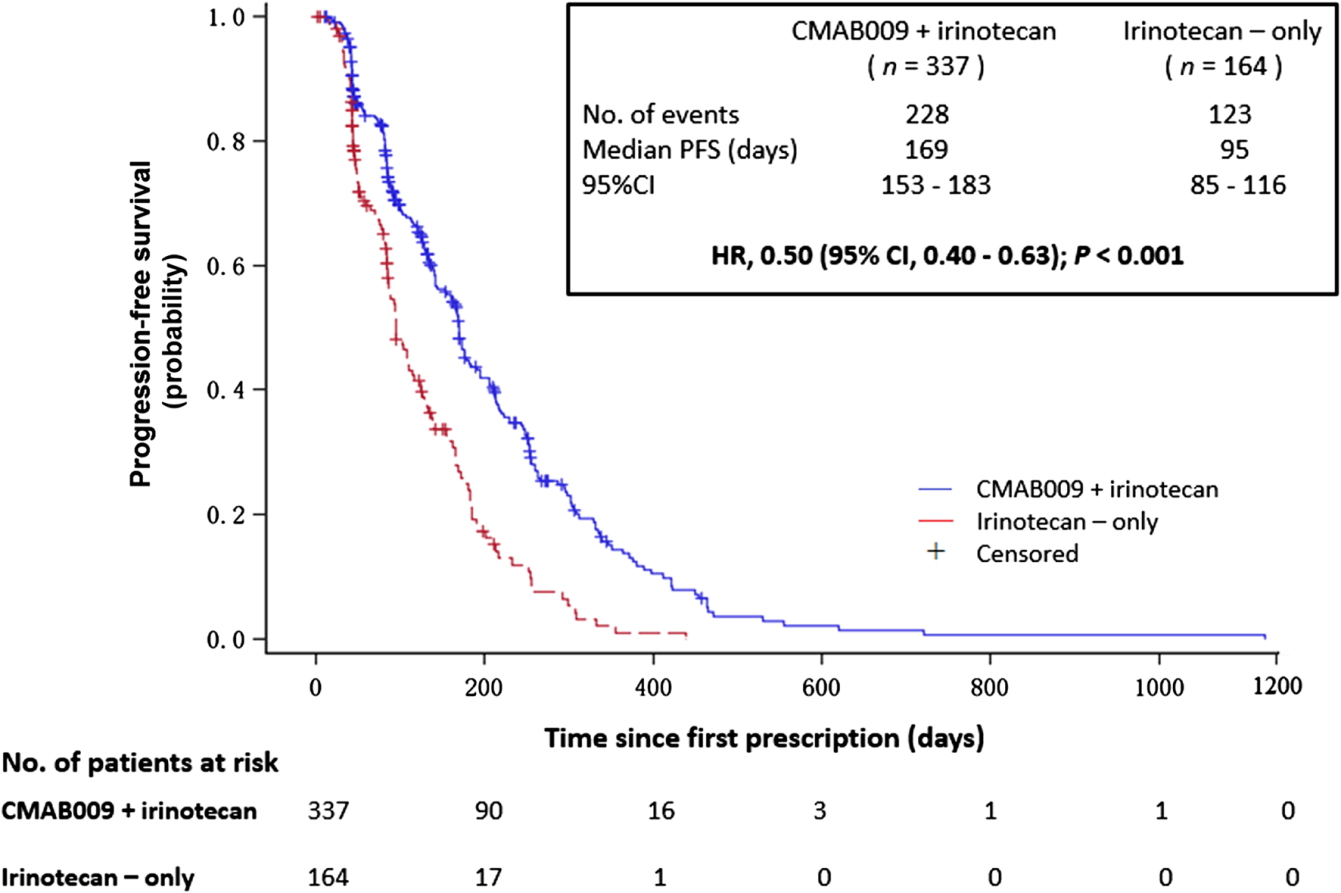
Kaplan-Meier analysis comparing the progression-free survival of patients from the CMAB009 plus irinotecan arm to those in the irinotecan-only arm only. PFS, progression free survival; HR, hazard ratio; CI, confidence intervals

The median PFS was significantly longer in the CMAB009 plus irinotecan arm than in the irinotecan-only arm (169 vs. 95 days; HR, 0.50; 95% CI 0.40 to 0.63; *P *< 0.001) (Fig. 2). In the sequential-CMAB009 arm, the median PFS was 84 days (95% CI 65–113 days).

#### Secondary endpoints

**Fig. 3:**
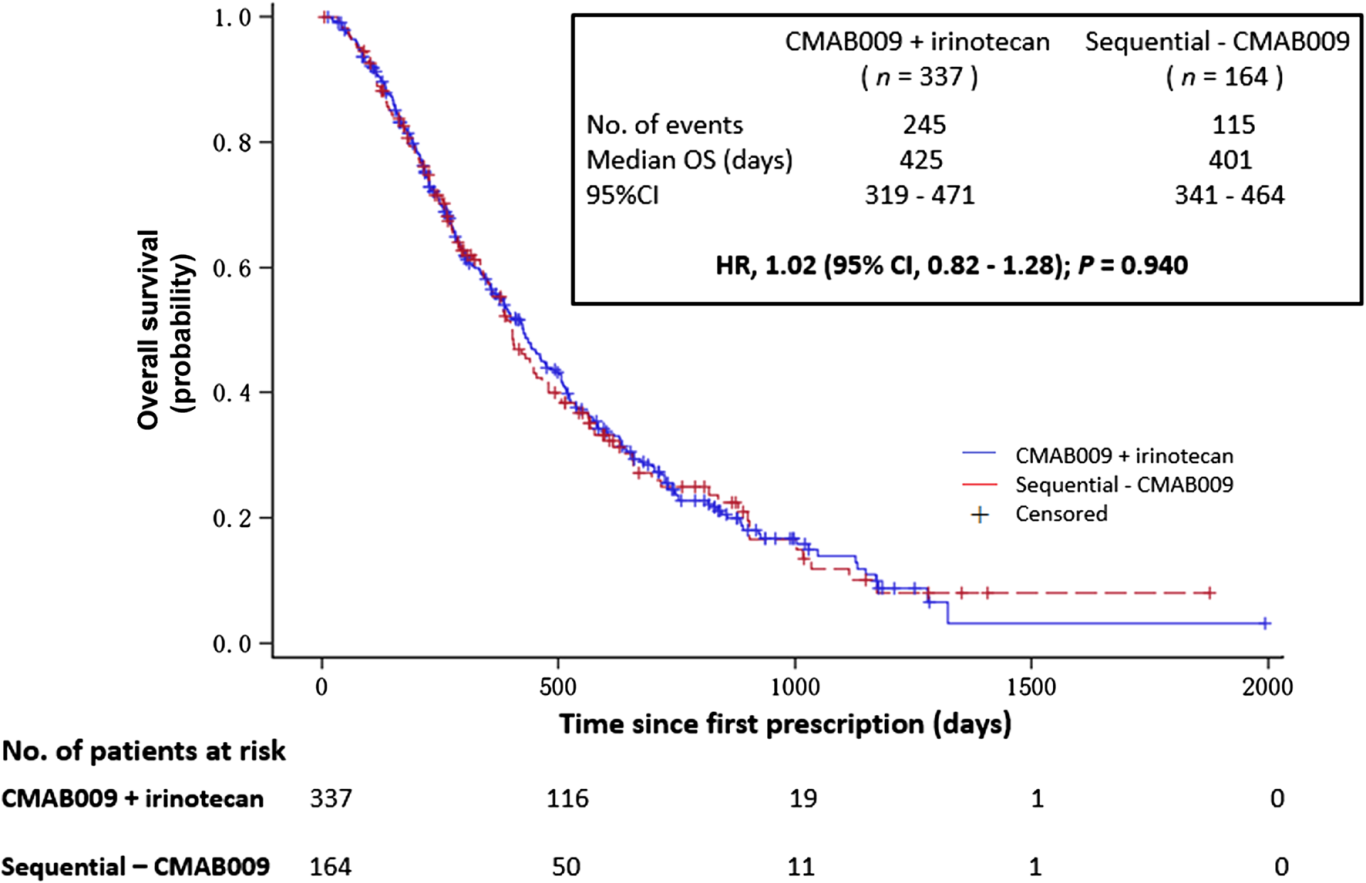
Kaplan-Meier analysis comparing the overall survival of patients from the CMAB009 plus irinotecan arm to those in the sequential-CMAB009 arm. OS, overall survival; HR, hazard ratio; CI, confidence intervals

Median OS was 425 days in the CMAB009 plus irinotecan arm and 401 days in the sequential-CMAB009 arm (HR, 1.02; 95% CI 0.82 to 1.28; *P *= 0.940) (Fig. 3). The DCR and CBR were higher for patients in the CMAB009 plus irinotecan arm as compared to the irinotecan-only arm (both *P  *< 0.001, Table 3). In the sequential-CMAB009 arm, the DCR and CBR were 63.5% and 23.1%, respectively.

**Fig. 4:**
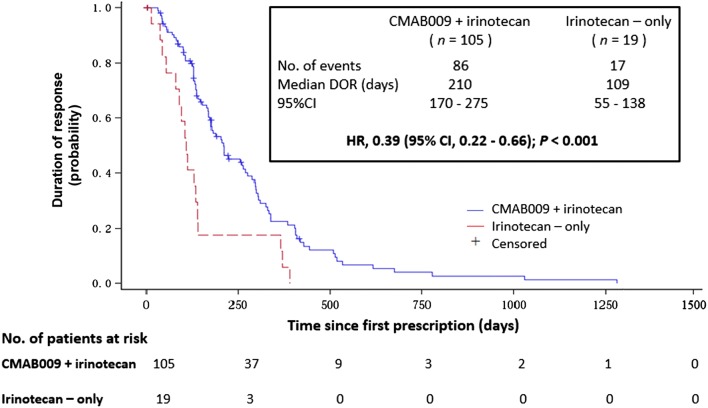
Kaplan–Meier analysis for the duration of treatment response. Median DOR was significantly longer in patients who received CMAB009 plus irinotecan compared with those who received irinotecan-only. DOR, duration of response

DOR in the CMAB009 plus irinotecan arm was almost twice of that in the irinotecan-only arm (210 vs. 109 days, HR, 0.39; 95% CI 0.22 to 0.66; *P *< 0.001; Fig. 4). For the sequential-CMAB009 arm, the DOR was 148 days (95% CI 59 to 230 days).

### Treatment safety

Over the course of the study, 96.2% (484/503) of patients experienced at least one adverse event. There were 55.3% (187/338) of patients in the CMAB009 plus irinotecan arm and 37.6% (62/165) of patients in the irinotecan-only arm who experienced at least one grade ≥ 3 adverse event. Although the frequency and severity of some adverse events were greater in the CMAB009 plus irinotecan arm, however, CMAB009 plus irinotecan was generally well-tolerated. The most common adverse events (Table 4) consisted of diarrhea (39.6% vs. 35.8%), emesis (18.9% vs. 37.0%), leucopenia (50.0% vs. 39.4%), neutropenia (30.5% vs. 19.4%), and fatigue (22.2% vs. 16.4%) in CMAB009 plus irinotecan and irinotecan-only arms, respectively. Hypersensitivity reactions were experienced in 0.9% (4/453) of patients in this study, three (one grade 1, one grade 2 and one grade 3) in the CMAB009 plus irinotecan arm and one (grade 2) in the sequential-CMAB009 arm (Table 4). As expected, the addition of CMAB009 to irinotecan significantly increased the risk of skin rash (66.9% vs. 5.5%, *P *< 0.001) and paronychia (9.8% vs. 0.0%, *P *< 0.001). Additionally, one patient in the sequential-CMAB009 arm developed grade 4 neutropenia but was determined unrelated to the CMAB009.

**Table 4:** Treatment-emergent AE occurring during the study in the safety analysis set

Parameters	CMAB009 plus irinotecan (*n* = 338)				Irinotecan-only (*n* = 165)			
	All grades grade		Grade 3/4		All grades grade		Grade 3/4	
	*n*	%	*n*	%	*n*	%	*n*	%
Any drug-related AE^a^	334	98.8	187	55.3	150	90.9	62	37.6
Diarrhea	134	39.6	35	10.4	59	35.8	12	7.3
Emesis	64	18.9	14	4.1	61	37.0	13	7.9
Leucopenia	169	50.0	54	16.0	65	39.4	15	9.1
Neutropenia	103	30.5	54	16.0	32	19.4	14	8.5
Fatigue	75	22.2	9	2.7	27	16.4	6	3.6
Special AE^b^								
Rash	226	66.9	22	6.5	9	5.5	1	0.6
Paronychia	33	9.8	4	1.2	0	0	0	0.0
Infusion reaction	2	0.6	1	0.3	1	0.6	0	0.0
Hypersensitivity reaction	3	0.9	1	0.3	1^c^	0.9^c^	0^c^	0.0^c^

AE, adverse events

^a^Includes cases having special adverse events

^b^Special adverse events were of categorized based on events that occurred in previous study and were reported for CMAB009 related-toxicities

^c^Sequential-CMAB009 arm (*n* = 115)

### Immunogenicity

A total of 1115 serum samples were obtained from 310 patients (249 patients from the CMAB009 plus irinotecan arm and 61 patients from the sequential-CMAB009 arm) and were analyzed for the presence of ADA, which was detected in only 3.6% (11/310) of patients. Excluding 4 patients with pre-existing ADA, the incidence of ADA in this study cohort was found to be 2.3% (7/310) (Table 5). All the ADA positive patients were in the CMAB009 plus irinotecan arm and none in the sequential-CMAB009 arm. Samples positive for binding antibodies in the confirmatory ADA assay were further evaluated for the presence of NAb to the CMAB009, of which 1.3% (4/310) patients were found to be positive (Table 5). No significant differences in clinical safety were found between ADA positive patients and ADA negative patients.

**Table 5:** Summary of the detection of ADA and NAb in the enrolled patients

Case number	The time of collection (Blood sample, weeks)	ADA	NAb
29	30	+	+
69	30	+	–
102	6	+	+
120	12	+	–
139	0	+^a^	+
139	12	+	–
193	6	+	+
234	6	+	+
242	12	+	–
438	0	+^a^	–
467	0	+^a^	+
510	0	+^a^	+

ADA, anti-drug antibodies; NAb, neutralizing antibodies

^a^Pre-existing ADA: ADA present in samples from treatment-naïve subjects or ADA in pre-dose (CMAB009) subject samples

## Discussion

This is the first prospective, open-label, randomized, phase III study comparing the clinical efficacy and safety of an anti-EGFR monoclonal antibody in *KRAS* wild-type mCRC patients with documented previous failure with FOLFOX regimen. In this study, we have found that patients treated with CMAB009 plus irinotecan demonstrated significantly better ORR and prolonged PFS as compared with those having irinotecan-only. In addition, the combination treatment with CMAB009 was generally well-tolerated and manageable. Therefore, this regimen could be considered as a new standard of treatment in the second-line setting for *KRAS* wild-type mCRC patients after failure with the FOLFOX regimen.

From the knowledge of the predictive value of *KRAS* mutation (codons 12 and 13) status for the efficacy of cetuximab, wild-type *KRAS* is required for evaluating cetuximab efficacy in mCRC patients [19–23]. *KRAS* mutations have been reported in 30% to 50% of CRC tumors and are also common in other tumor types [19]. In the present study, of the 1077 mCRC initially assessed in the *KRAS* analyses, of which the *KRAS* status in 35 patients could not be determined due to insufficient or poor-quality DNA samples, only 512 patients were found to have *KRAS* wild-type mCRC, demonstrating an observed *KRAS* mutations incidence of 32.3%. This was within the expected range of previous studies which reported mutation rates of approximately 36% [24–26].

Treatment with CMAB009 plus irinotecan, as compared to irinotecan-only, demonstrated significantly improved ORR, reduced the risk of disease progression by nearly 50% (PD, 13.9% vs. 26.8%, respectively), improved clinical efficacy (DOR, 210 days vs. 109 days; DCR, 80.1% vs. 65.2%; CBR, 30.0% vs. 14.6%, respectively), and had an acceptable safety profile. Further, the findings of this study showed that when CMAB009 was used as a third-line treatment in the sequential-CMAB009 arm, 13.9% (16/115) of the patients achieved PR with a PFS of 84 days, and 49.6% (57/115) of the patients achieved SD, comparable with the results of the CO.17 Trial [27] from the National Cancer Institute of Canada Clinical Trials Group (NCIC CTG), in which cetuximab was found to significantly improve PR (8%), SD (31.4%), and quality of life compared to the best supportive care in CRC patients in whom other treatments had failed. Several studies have reported the combination of irinotecan with other drugs, such as modified XELIRI (mXELIRI, capecitabine plus irinotecan) regimen and FOLFIRI (leucovorin, fluorouracil, and irinotecan), with or without bevacizumab regimens. The median OS for mXELIRI with or without bevacizumab was found to be non-inferior (16.8 months vs. 15.4 months) to FOLFIRI with or without bevacizumab regimens for mCRC [28]. A small-scale retrospective study reported that a re-challenge strategy with cetuximab and irinotecan may be active in patients with *RAS* and *BRAF* wild-type mCRC with acquired resistance to first-line irinotecan- and cetuximab-based therapy [29]. These results showed that CMAB009/cetuximab plus irinotecan might have good clinical efficacy in *KRAS* wild-type mCRC patients.

This study was also designed to explore whether CMAB009 plus irinotecan therapy would prolong OS. Our results showed that the median OS was similar between the CMAB009 plus irinotecan and sequential-CMAB009 arms (425 days vs. 401 days, *P *= 0.940), which we presume was possibly influenced by the sequential CMAB009 treatment in the patients after irinotecan failure, and thereby indicated that either CMAB009 plus irinotecan or sequential-CMAB009 may be considered as an effective treatment choice.

The safety profile of CMAB009 plus irinotecan in our study was comparable with that of other anti-EGFR monoclonal antibodies [27, 30–32]. Skin rash is associated with all EGFR inhibitors and is the most frequently associated with cetuximab/CMAB009. This adverse event seems to be closely linked to the biologic activity of cetuximab/CMAB009 as EGFR is expressed on the epidermal keratinocytes and hair follicles, and is thought to play a role in maintaining the skin integrity and follicular homeostasis [33]. Therefore blocking these effects may be responsible for the observed rashes. As such, in the present study, the most noticeable adverse event related with CMAB009 was skin rash (66.9%), which was similar to the cetuximab-related acneiform rash reported in 76.3% of patients in the EPIC study [20]. Notably, the CMAB009 plus irinotecan arm did not significantly increase gastrointestinal toxicity as compared with the irinotecan-only arm.

Humans have baseline levels of antibodies against certain non-human glycan motifs, including N-gl ycolylneuraminic acid (NGNA) and Gal (α 1-3) Gal, and severe hypersensitivity reactions occurring during the initial infusion of cetuximab are mediated by preexisting IgE antibodies against cetuximab [14, 34]. A high prevalence of severe hypersensitivity reactions of approximately 2% was reported in patients who had been injected with cetuximab because cetuximab is attached to N-linked oligosaccharide containing the Gal (α 1-3) Gal motif at the Fab region [16]. This non-human glycan may induce immunogenicity [35]. However, CMAB009 expressed in CHO and has a different glycosylation pattern not containing the NGNA or Gal (α 1-3) Gal motif at the Fab region [34, 36]. Therefore, CMAB009 has lower immunogenicity than cetuximab and the presence of ADA was found to be low at 3.6% (11/310). Only 0.9% (4/453) of patients experienced hypersensitivity reactions in our study, three in the CMAB009 plus irinotecan arm and one in the sequential-CMAB009 arm (Table 4).

Our study had several limitations worth noting. First, this study did not analyze other biomarkers such as NRAS. It was recently reported that NRAS was mutated in 6% of mCRC and were associated with a shorter OS compared to wild-type patients [31]. One meta-analysis showed that non-functional mutation or loss of NRAS, BRAF, PIK3CA, and PTEN predicted poor efficacy of cetuximab [31, 32]. Therefore, to demonstrate the predictive value of RAS and BRAF, we propose a prospective phase III study to explore the clinical efficacy and safety of CMAB009 plus FORFIRI as first-line chemotherapy in RAS/BRAF wild-type patients with mCRC in China. Second, there were some patients failing to provide serum samples and some samples could not be analyzed due to hemolysis. This is because the release of cellular material into the serum or plasma would have introduced additional confounding factors in the downstream analysis of such samples and were therefore excluded from the immunogenicity analysis. Third, the quality of life of the patients was not assessed.

## Conclusions

Treatment with CMAB009 plus irinotecan, compared to irinotecan-only, demonstrated superior clinical efficiency and was well tolerated as a second-line of treatment in *KRAS* wild-type mCRC patients with documented previous failure with the FOLFOX regimen. Therefore, this regimen could be considered as an optimal second-line treatment of choice for such patients. Further, for those whose disease progressed after being treated with irinotecan-only, as a second-line of treatment, switching to CMAB009 can be considered as an effective third-line of treatment.

## Data Availability

The datasets obtained and analyzed during the present study are available from the corresponding author on reasonable request.

## Abbreviations

ADA: anti-drug antibodies
CBR: clinical benefit rate
CRC: colorectal cancer
CHO cells: Chinese hamster ovary cells
CapeOX: capecitabine plus oxaliplatin
DCR: disease control rate
DOR: duration of response
EGFR: epidermal growth factor receptor
5-FU: 5-fluorouracil
FOLFOX: 5-fluorouracil (5-FU)/leucovorin (LV) plus oxaliplatin
LV: leucovorin
mCRC: metastatic colorectal cancer
CMAB009: novel recombinant human/mouse chimeric epidermal growth factor receptor (EGFR) monoclonal antibody
ORR: overall response rate
OS: overall survival
PFS: progression-free survival
